# Supplementary material for: Effect of high-dose β-Alanine supplementation on uphill cycling performance in World Tour cyclists: A randomised controlled trial
Source: PLoS One. 2024 Sep 3;19(9):e0309404. doi: 10.1371/journal.pone.0309404 (PMC11371202; doi:10.1371/journal.pone.0309404)
Supplement: S2 File — (PDF) [file pone.0309404.s003.pdf]

A RANDOMISED DOUBLE-BLIND CLINICAL TRIAL TO ANALYSE THE EFFICACY OF SHORT-TERM β-ALANINE SUPLEMENTATION IN PERFORMANCE OUTCOMES OF ROAD PROFFESIONAL ENDURANCE CYCLISTS.

1. TITLE..3

2. OBJECTIVES..3

3. TRIAL DESIGN 3

4. PRODUCT SPECIFICATIONS 3

5. PARTICIPANTS 4

6. WITHDRAWAL AND SUBJECT REPLACEMENT..4

7. REMOVAL CRITERIA 4

8. RANDOMISATION OF PARTICIPANTS 5

9. BLINDING..5

10. STUDY DESIGN...5

11. STUDY VARIABLES..8

12. STUDY DEVEOLPMENT 9

13. STATISTICAL ANALYSIS..10

14. ETHICS 10

1. TITLE

RANDOMISED CLINICAL TRIAL TO ANALYSE THE EFFICACY OF SHORT-TERM β-ALANINE SUPLEMENTATION IN PERFORMANCE OUTCOMES OF ROAD PROFFESIONAL ENDURANCE CYCLISTS.

2. OBJECTIVES

Main objective:

To analyse the efficacy of short-term β-alanine supplementation to performance outcomes in professional cyclists.

Secondary objectives:

- Analyse contribution of the product to participants' fatigue.
- Analyse product's effect during one week to performance variables in 2 metabolic mixed trials.

3. TRIAL DESIGN

A double-blind, placebo-controlled, randomised, with two arms (product and placebo) unicentric clinical trial to analyse the efficacy on physical performance parameters of a product designed for sport during a lapse of one week (7 days).

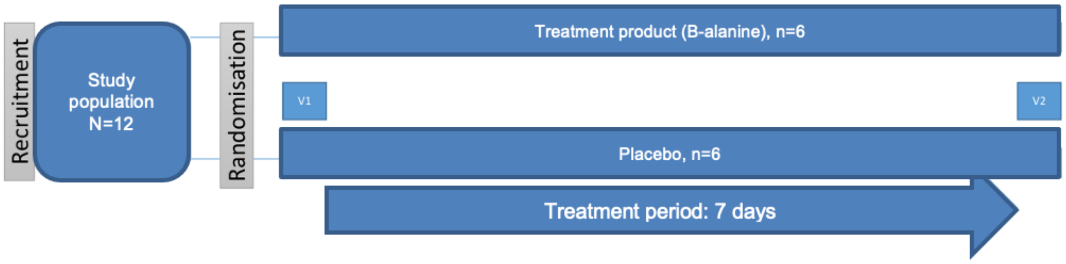

Figure 1: Study design and main variables.

4. PRODUCT SPECIFICATIONS

Product: β-alanine (MARNYS®)

Supplementation strategy: 5 g, four times a day with main meals, during 7 days after initial assessment, and until the end of the study.

5. PARTICIPANTS

A total of 12 subjects will participate in the study, complying with all the inclusion criteria and none of the exclusion criteria.

Inclusion criteria:

In order to be included as participant, the following criteria must be met:

- Male, professional cyclist.
- Start the study well rested.

Exclusion criteria:

- Chronic disease.
- Had suffered an injury which impeded training during the month before commencement of the study.
- Not understanding or refusing to sign the written consent prior the study.
- Previous β-alanine supplementation withing the two months before commencing the study.

6. WITHDRAWAL AND SUBJECT REPLACEMENT

Participants can leave the study at any moment, with or without reason and without further consequences. Furthermore, they won't be followed-up of substituted. In case should a withdrawal happen, reason will be recorded. Conversely, the investigation team have the power of discretion to remove a subject if considered necessary. Data obtained from these subjects will be conserved and included in the global analysis.

7. REMOVAL CRITERIA

In case the subject is not able to comply with all requirements of any of the procedures is harmful for him, it will be removed from the study.

The following requirements must be meet before the physical trials:

- Not respecting rest the day before physical tests.
- Not being in a fasted state (at least 2 h from last meal).

- Having consumed stimulants acutely before the trial, or under drug treatment affecting perceived effort of the trial.
- Not being available to perform every trial on same conditions at the same time of the day.
- Not sticking to the same diet, 24 h before each trial.

Exclusion criteria which justifies removal of a participant includes:

- Adverse event
- Protocol violation
- Lost to follow-up
- Allergy to any of the components of the treatment or placebo product. This includes allergy to β-alanine, wheat, soy, nuts (including peanuts), sesame and its byproducts.

8. RANDOMISATION OF PARTICIPANTS

After recruitment, subject will be randomised and allocated to one group of the 2 study arms: treatment group or control group (placebo).

A simple randomisation will be performed using software by a random number generator, and assigned to participants.

9. BLINDING

Subjects are unaware of the study arm they belong to, so both products are identical in appearance, consisting the placebo of inactive substances. Both products will be manufactured by the same company and labeled for proper identification including participant number and trial code. Staff participating in the study are also blinded so they don't know which product is the treatment or placebo product.

10. STUDY DESIGN

For performance outcome analysis, two time-trials (TT) will be employed: One consisting of a time based TT (10-minute) on ergometer, which will conducted in an enabled room

in the host hotel of the cycling team, and one field distance-based TT of a track (Coll de Rates – 5.1 km)(Figure 1) for which subject are previously familiarized.

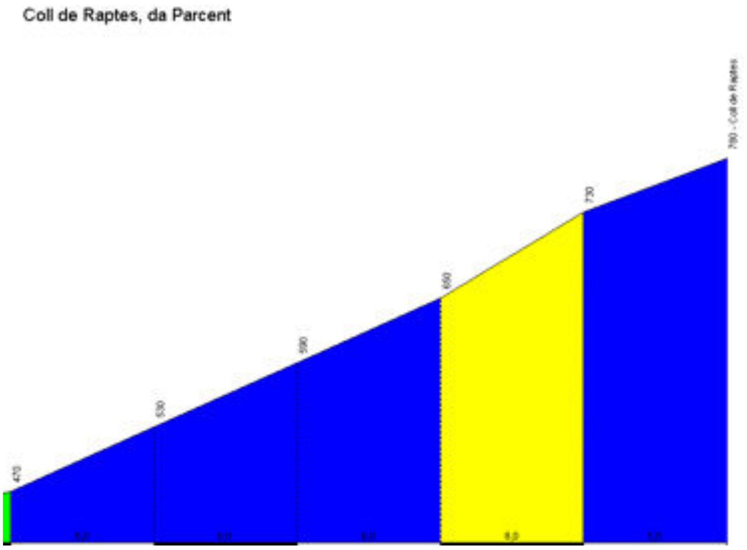

Figure 2: Elevation profile of the Coll de Rates track. Estimated distance is of 5.1 kilometers with a total ascent of 350 meters (6% inclination).

Trials and measurements:

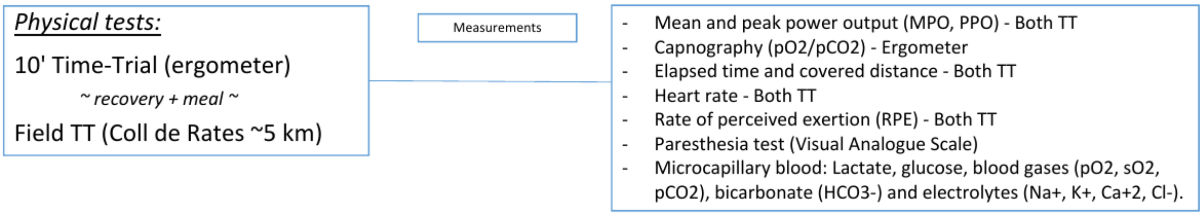

PHASE 1. Baseline measurements. (Day 1).

First visit (V1). Subjects must come in a fasted state, properly hydrated. Before commencement of trials, a blood sample from the antecubital vein will be withdrawn for amino-acid analysis. A 10-minute TT will be performed on an ergometer (Cyclus 2) using participant's own bike, to assess baseline performance variables. A pre-competitive warm-up will be performed before the trial. During the trial, physical plate and sprockets will remain fixed, allowing the participant to change their speeds virtually (through the ergometer control panel). Subjects must perform at maximum intensity for 10' at a pace that enables them to perform the maximum distance.

During this test, mean power output, peak power output, and distance covered will be measured as performance variables. Physiological variables will be measured during the trial including heart rate (chest band), partial pressure of O_2/CO_2, oxygen saturation ($SatO_2$) and pulsate index At the beginning, end, and 3' after the trial, microcapillary blood samples (70 µl) will be withdrawn, as well as a rate of perceived exertion (RPE) test (Borg test).

After this first TT, a controlled rest with a predefined meal designed by a nutritionist is given before the second "on field" TT. This consists of a constant ascent 5.1 km track (total ascent 350 m with 6% of inclination). After precompetitive warm-up (same as in the previous trial), subjects must complete the Coll de Rates track in the least time possible with a free pedaling cadence and gear ratio. During the trial, pedaling cadence (cadence sensor), speed and distance (hub speed sensor), power output (power pedals), GPS trace (map positioning, estimated speed, estimated distance, and elapsed time) and heart rate are registered through personal computer/sportwatch that must be provided to researchers for further analysis. Biochemical microcapillary samples will be taken before and after the test and 3' thereafter, as well as RPE and further analysed by gas analyser (ABL90FLEX).

After the TT's both placebo and treatment product are given to participants for starting the 12-day supplementation program.

PHASE 2. Final measurement. (Day 7).

Second visit (V2). Same tests are repeated, and same biochemical samples as in V1.

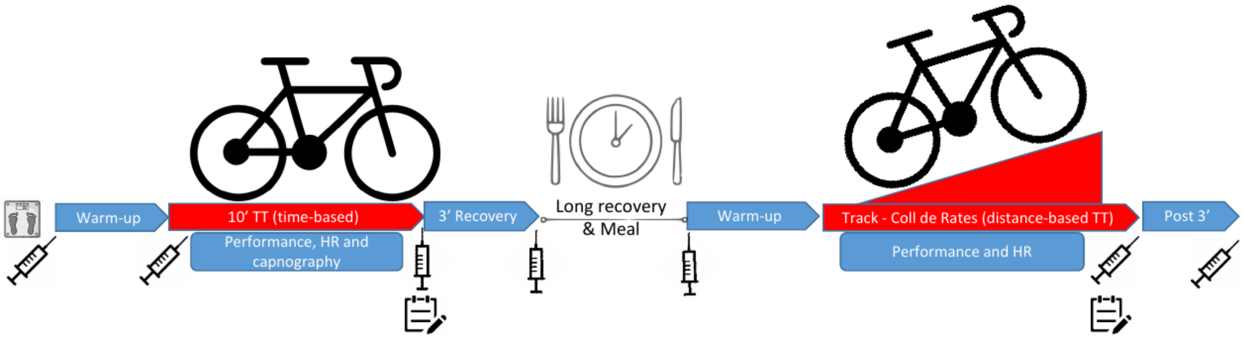

Figure 3: Overview of each visit schedule.

11. STUDY VARIABLES

The following variables will be measured for the study.

- **Demographic and clinical characteristics**
  - Age (years)
  - Years of experience (years)
  - Previous experience with beta-alanine (Yes or No)
  - Weight (kg)
  - Height (cm).
  - Body composition (bioimpedianciometry provided by weight scale)
  - Amino acid blood homeosthasis (L-histidine and β-alanine)
  - VO_2max (ml/min/kg)*
  - Maximum power output (Watts)*
  - Power output at ventilatory threshold 2 (Watts)*

  *Ideally provided by last previous incremental maximal tests performed elsewhere prior the study. Date of the test should be given.

- **Fatigue:**
  - **Rate of perceived exertion (RPE)**. **Borg Test**.

    Subjects are asked to visually mark a discrete scale from 0-10 (corresponding the highest mark to the highest effort perceived) at the end of each TT.

- **Performance outcomes:**

  Physical performance is measured by direct variables: mean power output (MPO) and peak power output by ergometer (Cyclus 2) and/or pedal power sensors in for the time-based TT, together with cadence. In the filed distance-based TT, mean and peak power will be measured by pedal power sensors, cadence by personal cadence sensor, speed and distance by hub speed sensor and time to complete the trial by GPS tracker and chronometer (double-check).
- **Paresthesia test**

  Measured as a visual analogue scale (1-10) on the first and last intakes. A continuous line is displayed in a paper in which subjects must mark with a vertical line the highest perceived paresthesia (peak paresthesia) in the time lapse of 4 hours since the first intake. If no paresthesia is perceived the line must be marked on the vertical line of the scale.
- **Physiological responses:**
  - Heart-rate by chest-band during the trials and during the recovery phase.

12. <u>STUDY DEVEOLPMENT</u>

After recruitment, participants must sign the written consent prior to data gathering. A researcher will inform about risks and benefits of taking part in the experiment and is free to ask about any question to this respect. A total of 2 visits will be carried during the study, to the host hotel of participants.

Pre: Demographic data of the subjects, as well as informed consent are gathered.

First visit (Day 1): 10’ TT, recovery interval with standardised meal and Coll de Rates track (5.1 km). The product is given to participants.

Second visit (Day 7): Procedures from the same visit are repeated.

Step/Visit	V1	V2
**Clinical history**	+	
**Inclusion/exclusion criteria check**	+	
**Written consent**	+	

**Randomisation**	+	
**Product given to participants**	+	
**10’ TT (time-based TT)**	+	+
**5.1 km TT (distanced-based field TT)**	+	+
**Fatigue scale (RPE: Borg’s scale)**	+	+
**Performance measurements**	+	+
**Intra-test measurements**	+	+
**Physiological variables**	+	+
**Paresthesia test**	+	+

13. STATISTICAL ANALYSIS

Descriptive analysis.

Statistical descriptive data will be given of both study groups and as a global.

Continuous variables will be displayed as central tendency (mean) with dispersion (standard deviation). Categorical variables are showed as frequency table both as absolute and relative values.

In order to test group homogeneity, baseline characteristics of both are compared and showed in tabular form. Statistical test will be applied according to prior checking of data type. Usually, categorical variables are compared by a Chi-square test, and continuous variables by Student t-test (if assumptions are met).

Comparative analysis of variables

To analyse the product influence of the product to performance and fatigue variables between the two groups, a previous repeated measures analysis of variance is performed (rANOVA) with one intra-subject factor (placebo or product) and inter-subject (time). For post-hoc analysis, a Turkey or Bonferroni test will be employed. Comparison between significant effects will be performed.

14. ETHICS

Study will be carried out following the good clinical practices, and according to the declaration of Helsinki regarding human clinical trials.

Ethic committee assessment

According to general rules in research, the study must be approved by an ethic committee, in this case to the Ethics Committee of the Catholic University of Murcia (UCAM). This committee will also approve the written consent.
