# Supplementary material for: Effect of high-dose β-Alanine supplementation on uphill cycling performance in World Tour cyclists: A randomised controlled trial
Source: PLoS One. 2024 Sep 3;19(9):e0309404. doi: 10.1371/journal.pone.0309404 (PMC11371202; doi:10.1371/journal.pone.0309404)
Supplement: Attachment — Submitted filename: AUTHORS REPLY R2.docx [file pone.0309404.s005.docx]

**AUTHORS REPLY**

**A: First of all, we would like to express our gratitude to Reviewer #1 for providing us with comments and suggestions to help improve the quality of our paper. We have answered your comments point-by-point below and have marked our changes in red in the new version of the manuscript.**

Reviewer #1:

Major revision: My prior comment concerning using the appropriate statistical testing method based on the distribution of the data has not been adequately addressed. The sample sizes are too small to test for normal distributions; therefore, nonparametric tests are called for.

Prior Comment: A comprehensive reanalysis is required.

Since sample sizes of 5 and 6 are too small to be tested for a normal distribution, use nonparametric tests for the analysis, i.e., use the Wilcoxon rank sum test instead of the t-test. Furthermore, summarize data using medians and first and third quartiles instead of means and standard deviations.

**A: We thank the reviewer for the comment. Following the recommendation, we have implemented a new non-parametric analysis and modified the entire document accordingly.**

Minor revision:

Line 101: For the power justification, indicate if the t-test was the test used for the "comparisons of means test".

**A: Thank you for the comment. We have added the information. As the reviewer points out, a t-test is the test used.**

**A: First of all, we would like to express our gratitude to Reviewer #2 for providing us with comments and suggestions to help improve the quality of our paper. We have answered your comments point-by-point below and have marked our changes in red in the new version of the manuscript.**

Reviewer #2:

The text calls out Figure 1 to represent study flow but the attached figure 1 is the CONSORT Diagram.

**A: Modified.**

Please provide the manufacturer information for the bikes used.

**A: Added.**

The figures are still very blurry. The axes and legends cannot be read.

**A: The figures have been updated for better readability.**

All other comments have been sufficiently addressed. Some responses to the reviewers should be reflected in the manuscript text and not just in the responses to reviewer comment document.

**A: Thank you very much for the thorough review. We have included the information in the text.**

**A: First of all, we would like to express our gratitude to Reviewer #3 for providing us with comments and suggestions to help improve the quality of our paper. We have answered your comments point-by-point below and have marked our changes in red in the new version of the manuscript.**

Reviewer #3:

Thank you to the authors for the answers. The protocol used is correct, and the overall explanation of the findings is clear. However, I suggest checking these two articles for further improvement of the FTP concept.

Wong S, Burnley M, Mauger A, Fenghua S, Hopker J. Functional threshold power is not a valid marker of the maximal metabolic steady state. J Sports Sci. 2022 Dec;40(23):2578-2584. doi: 10.1080/02640414.2023.2176045. Epub 2023 Feb 20. PMID: 36803419.

Vinetti G, Rossi H, Bruseghini P, Corti M, Ferretti G, Piva S, Taboni A, Fagoni N. Functional Threshold Power Field Test Exceeds Laboratory Performance in Junior Road Cyclists. J Strength Cond Res. 2023 Sep 1;37(9):1815-1820. doi: 10.1519/JSC.0000000000004471. Epub 2023 Feb 2. PMID: 36692223; PMCID: PMC10448799.

**A: Thank you very much to the reviewer for their comment. We have carefully read the articles you indicated. For the next study, we will take into account the aspects related to FTP mentioned in the provided studies.**
